# Impact of smoking status on incident hypertension in a Japanese occupational population

**DOI:** 10.1038/s41440-024-01996-x

**Published:** 2024-11-08

**Authors:** Ikumi Yamato, Yasuo Kansui, Kiyoshi Matsumura, Minako Inoue, Ai Ibaraki, Satoko Sakata, Hisatomi Arima, Kenichi Goto, Takanari Kitazono

**Affiliations:** 1 Department of Medicine and Clinical Science, Graduate School of Medical Sciences, Kyushu University, Fukuoka, Japan; 2 Kitakyushu Wakasugi Hospital, Fukuoka, Japan; 3 Center for Cohort Studies, Kyushu University, Fukuoka, Japan; 4 Department of Epidemiology and Public Health, Kyushu University, Fukuoka, Japan; 5 Department of Preventive Medicine and Public Health, Fukuoka University, Fukuoka, Japan; 6 Department of Health Sciences, Kyushu University, Fukuoka, Japan

**Keywords:** Incident hypertension, Smoking cessation, Tobacco smoking

## Abstract

Hypertension and tobacco smoking pose independent risks for cardiovascular diseases, but their association is unclear especially in Japanese. We investigated the impact of smoking status on the risk of new-onset hypertension in male and female Japanese workers. We evaluated 5439 subjects without hypertension who participated in medical check-ups in 2007–2018. The outcome was the development of hypertension (blood pressure ≥140/90 mmHg or initiation of antihypertensive drugs). Cox’s proportional hazards models were used to assess the association between smoking status and the hypertension incidence. During the average 6.0-year follow-up, 1395 individuals (25.6%) developed hypertension. The crude incidence rates of hypertension (per 100 person-years) were: current non-smokers (*n* = 3033), 3.4; quitters (*n* = 445), 4.2; and sustained smokers (*n* = 1961), 5.7. The multivariable-adjusted hazard ratio was 1.34 (1.20–1.50) for sustained smokers and 1.03 (0.86–1.24) for quitters compared to current non-smokers (P for trend <0.01). In stratified analyses, the risk of incident hypertension was significantly higher in the sustained smokers with lower blood pressure or without diabetes at baseline versus the current non-smokers. A significant risk reduction of hypertension development due to smoking cessation was revealed in the subjects with higher blood pressure levels at baseline or without body weight gain after smoking cessation. Smoking was an independent risk factor for incident hypertension. Smoking cessation reduced the risk of hypertension development compared to sustained smoking, especially among the subjects with higher blood pressure levels. Maintaining one’s body weight after smoking cessation would also help prevent hypertension development.

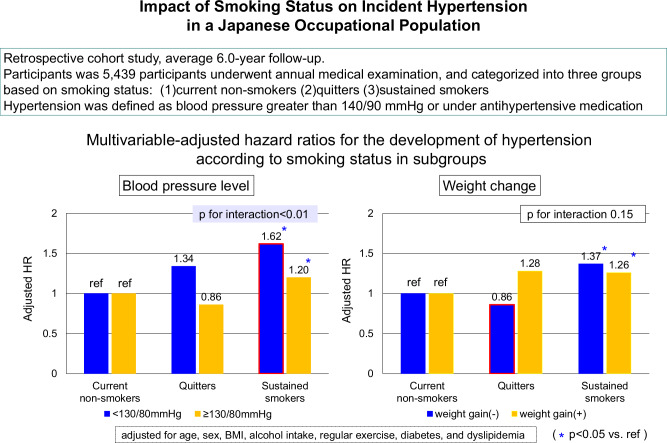

## Introduction

Hypertension has been confirmed as one of the most important risk factors for cardiovascular disease, and its estimated global prevalence in 2025 will be 1.56 billion [1]. Tobacco cigarette smoking has also been shown to be a serious risk factor for cardiovascular disease [2], and several countries’ relevant guidelines strongly recommended that individuals should avoid or quit smoking tobacco products in order to reduce their risks of cardiovascular disease [3–6]. However, the precise relationship between smoking status and the development of hypertension has not yet been established.

Although it is known that one of the acute effects of tobacco smoking is a ≥ 15-min increase in blood pressure due to activation of the sympathetic nervous system [7, 8], the effects of long-term cigarette smoking on changes in blood pressure are not completely clear [9]. Population-based studies have indicated that cigarette smoking was independently associated with an elevated risk of the development of hypertension [10–13]. However, other studies observed lower blood pressure levels among smokers [14–17]. These findings suggest that, despite extensive studies, the results were inconsistent and were not sufficient in concluding the effects of smoking on incident hypertension especially in Japanese.

Furthermore, the effects of tobacco smoking cessation on blood pressure have also not been determined. Smoking cessation has been reported to result in a 4 to 5 kg weight gain [18, 19], and there is concern that weight gain after smoking cessation may undermine the benefits of smoking cessation [20]. Previous studies evaluating the harms associated with weight gain after smoking cessation have focused exclusively on cardiovascular disease or type 2 diabetes [21–23], and the literature on the association between weight gain after smoking cessation and the development of hypertension is particularly limited in the Japanese population. Since a recent Japanese study reported that weight gain after smoking cessation is associated with future development of hypertension [24], it is important to clarify the association between smoking status and the development of hypertension considering body weight changes during an observation period.

We therefore conducted the present study to determine the chronic effects of tobacco smoking on the incidence of hypertension in Japanese young and middle-aged workers. We focused on this younger population because is particularly important to prevent cardiovascular diseases in their later years. We also investigated the relationship between smoking cessation and incident hypertension with a focus on the baseline blood pressure level and changes in body weight after smoking cessation.

Point of view

- Clinical relevance
  Tobacco smoking was associated with incident hypertension. Managing weight gain would be important to reduce the risk of developing hypertension after smoking cessation.
- Future direction
  Further studies are necessary to determine whether the relationship between smoking status and the development of hypertension is also found in the elderly, women, and other ethnicities.
- Consideration for the Asian population
  Asian countries, particularly among men, still have high tobacco smoking rates compared to Western countries. In order to reduce smoking-related diseases including hypertension in Asia, maintaining an appropriate body weight after smoking cessation might also be important.

## Subjects and methods

### Study design and subjects

**Fig. 1:**
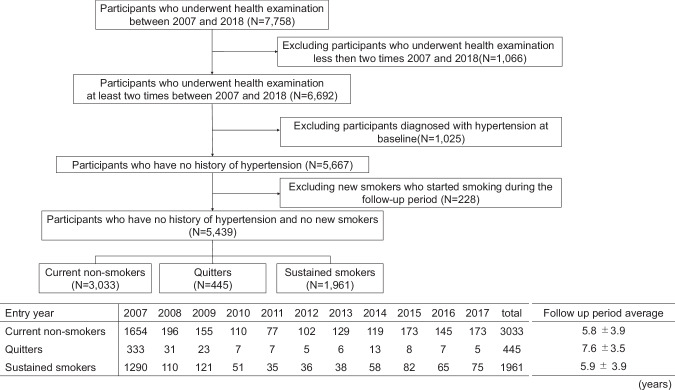
Flowchart and table showing the study participant enrollment. The table shows the baseline health examination year and the number of participants for each smoking status, and the average follow-up years for each group

The study group consisted of 7758 employees (≥18 years old) of a railway/bus company in Japan who participated in any of their employer’s annual medical check-ups over the years from 2007 to 2018. Individuals whose follow-up period was <1 year (*n* = 1066), those who already had hypertension (*n* = 1025), and those who began smoking during the observation period (*n* = 228) were excluded from the study. The remaining 5439 individuals were enrolled (Fig. 1). The study protocol was approved by the Ethics Committee of Kyushu University, and the requirement for written informed consent was waived in light of the study’s retrospective design and the anonymization of the subjects’ data.

### Data collection

At the baseline examination and during the study period, each subject’s sitting blood pressure was measured at the right upper arm by a sphygmomanometer after 5 min of rest. Low-density lipoprotein cholesterol (LDL-C), high-density lipoprotein cholesterol (HDL-C), triglyceride, glucose, and HbA1c were measured with the use of casual (non-fasting or fasting) blood samples. The serum triglyceride, HDL-C, and LDL-C levels were determined enzymatically. The serum glucose and HbA1c levels were measured using the hexokinase-UV method and latex aggregation method, respectively. The HbA1c level (%) was converted to a National Glycohemoglobin Standardization Program (NGSP) equivalent value using the following formula: HbA1c (NGSP value) = HbA1c (Japan Diabetes Society [JDS] value) + 0.4 [25].

The estimated glomerular filtration rate (eGFR) was defined using the formula provided by the Japanese Society of Nephrology: eGFR (mL/min/1.73 m^2^) = 194 × age^−0.287^ × serum creatinine^−1.094^ (×0.739 if female) [26]. The body mass index (BMI) was defined as weight (in kg) divided by height (in m^2^). Information on each subject’s medical history, habitual alcohol intake, and current tobacco-smoking status was obtained by interview or questionnaires. Drinking habits was classified as either current or not. Regular exercise was defined as an exercise habit of ≥30 min per day more than two times per week.

### Follow-up and outcome

During the follow-up period from 2007 to 2018, we defined the baseline of each participant as the first time when each participant had an annual health check-up, and then we followed him/her up to 2018. The outcome was the incidence of hypertension (systolic blood pressure (SBP) ≥ 140 mmHg, diastolic blood pressure (DBP) ≥ 90 mmHg, or the use of antihypertensive drugs).

### Definitions of diabetes mellitus, dyslipidemia, overweight, and higher blood pressure

Dyslipidemia was defined as HDL-C < 40 mg/dL, triglyceride ≥150 mg/dL, LDL-C ≥ 140 mg/dL, or current use of lipid-lowering agents. Diabetes was defined as a fasting serum glucose level at ≥126 mg/dL, a casual serum glucose level ≥200 mg/dL, an HbA1c level ≥6.5% or the use of an antihyperglycemic agent. Overweight/obese was defined as BMI of ≥23 kg/m^2^ [27]. Higher blood pressure was defined as SBP ≥ 130 mmHg or DBP ≥ 80 mmHg according to the Japanese Society of Hypertension Guidelines for the Management of Hypertension 2019 (JSH 2019) [5]. Weight gain was defined as a ≥ 3-kg increase from the subject’s baseline value to the end of study.

### Statistical analyses

We divided the subjects into the following three categories based on their smoking status at the beginning of the study: (1) the current non-smokers (*n* = 3033) were the subjects with no history of smoking at baseline or during the follow-up period (*n* = 2037) or with only a past history of tobacco smoking at baseline (*n* = 996); (2) the quitters (*n* = 445) were the subjects who were smoking at the beginning of the study but stopped smoking during the follow-up period and maintained their smoking cessation; and (3) the sustained smokers (*n* = 1961) were the subjects with a history of tobacco smoking not only at study entry but also during the follow-up period.

We used analysis of variance and chi-square test to compare continuous and categorical variables, respectively, among smoking status in baseline characteristics. We used the person-year approach to calculate the crude incidence rates of hypertension. Age-sex adjusted and multivariable-adjusted hazard ratios (HRs) and 95% confidence intervals (CIs) were estimated using Cox’s proportional hazards models. Multivariable logistic regression analysis was also used to estimate odds ratio with 95% confidence intervals for the development of hypertension. The heterogeneities of smoking status on the development of hypertension were compared among the subgroups divided by other risk factors (age, sex, overweight, weight gain, higher blood pressure, exercise habit, alcohol intake, dyslipidemia, and diabetes) by adding an interaction term to the statistical model. A two-tailed *p* < 0.05 was considered statistically significant. Missing values were processed using the pairwise deletion method. This allowed the analysis to be performed using valid data even when missing data were available. All data analyses were carried out using SAS ver. 9.4 (SAS Institute, Cary, NC, USA).

## Results

The subjects’ baseline clinical characteristics are summarized in Table 1. Compared to the current non-smoker group, the sustained smoker and quitter groups were each significantly older and had significantly larger proportions of males. The values of SBP and DBP, pulse rate, serum LDL-C and serum triglyceride were significantly higher in the sustained smokers and the quitters compared to those in the current non-smokers. Regular alcohol intake was significantly more frequent in the sustained smoker and quitter groups compared to the current non-smoker group.

**Table 1:** Baseline characteristics of the 5439 Japanese workers according to their smoking status

	Current non-smokers *n* = 3033	Quitters *n* = 445	Sustained smokers *n* = 1961
Men, *n* (%)	2189 (72)	404 (91)*	1852 (94)*
Age, yrs	36.1 ± 12.0	38.1 ± 9.7*	39.5 ± 9.9*
BMI, kg/m^2^	22.5 ± 3.2	22.9 ± 3.1	23.3 ± 3.3*
BMI ≥ 23 kg/m^2^, *n* (%)	1183 (39)	198 (44)	1003 (51)*
Weight change during study period, kg	1.1 ± 4.1	3.1 ± 5.1*	1.4 ± 4.3*
Weight gain during study period, *n* (%)	815 (27)	207 (47)*	590 (30)
No. of cigarettes/days	–	16.8 ± 8.5	18.7 ± 7.8
Smoking duration, yrs	–	17.6 ± 10.4	19.9 ± 10.0
SBP, mmHg	114 ± 12	116 ± 11*	117 ± 11*
DBP, mmHg	69 ± 11	72 ± 9*	73 ± 9*
Pulse rate, beats/min	69 ± 11	73 ± 12*	73 ± 11*
Alcohol intake, *n* (%)	1515 (50)	288 (65)*	1207 (62)*
Regular exercise, *n* (%)	1017 (34)	114 (26)*	499 (25)*
Diabetes, *n* (%)	81 (2.7)	17 (3.8)	80 (4.1)*
HbA1c, %	5.4 ± 0.5	5.4 ± 0.5	5.5 ± 0.6*
Hyperlipidemia, *n* (%)	1041 (34)	206 (46)*	1007 (51)*
Serum HDL-C, mg/dL	62 ± 14	57 ± 15*	55 ± 14*
Serum LDL-C, mg/dL	111 ± 30	115 ± 34*	117 ± 31*
Serum TG, mg/dL	116 ± 95	148 ± 114*	160 ± 133*
eGFR, mL/min/1.73 m^2^	91 ± 17	93 ± 15	91 ± 15

Values are mean ± SD or as number (*n*) (%)

*BMI* body mass index, *DBP* diastolic blood pressure, *eGFR* estimated glomerular filtration rate, *HDL-C* high-density lipoprotein cholesterol, *LDL-C* low-density lipoprotein cholesterol, *SBP* systolic blood pressure, *TG* triglycerides

*p < 0.05 vs. the Current non-smokers

In contrast, the serum HDL-C levels were significantly lower and proportion of subjects with an exercise habit was smaller among the sustained smokers and quitters compared to those among the non-smokers. The weight changes from the baseline to the end of study period was largest for quitters at 3.1 kg, who increased their weight by 0.3 kg from baseline to the last visit before smoking cessation, after that, gained an additional 2.8 kg. The eGFRs did not differ significantly among the groups.

The follow-up annual health check-up was averaged 5.6 times, and the average period of follow up was 6.0 ± 3.8 years. Current non-smokers, sustained smokers, and quitters were followed for 5.8, 5.9, and 7.6 years, respectively, until they developed hypertension or were censored. The follow-up period for quitters was significantly longer compared to the other groups. Timing of smoking cessation in quitters was averaged 3.9 years after study participation. After smoking cessation, quitters were followed until end of study period (average 3.7 years). During the follow-up duration, 1395 (25.6%) of the subjects developed new-onset hypertension. The incidence rates and hazards ratios for the development of hypertension are shown in Table 2. The crude incidence (per 100 person-years) of hypertension was 3.4 for the current non-smokers, 4.2 for the quitters, and 5.7 for the sustained smokers. The Cox hazard ratio (95% confidence interval [CI]) for the development of hypertension was 1.34 (1.20–1.50) for the sustained smokers and 1.03 (0.86–1.24) for the quitters compared to the non-smokers after the multivariable adjustment (model 1: age, sex, BMI, alcohol intake, regular exercise, diabetes mellitus, and dyslipidemia). These associations remained significant even after additional adjustment for SBP at baseline (model 2) or weight changes during the study period (model 3).

**Table 2:** Incidence rates and hazard ratios for the development of hypertension according to smoking status

	Current non-smokers *n* = 3033	Quitters *n* = 445	Sustained smokers *n* = 1961	P for trend
No. of events/person-years	595/17,530	143/3385	657/11,538	
Crude incidence rate per 100 person-years	3.4	4.2	5.7	
Age-sex adjusted HR (95%CI)	1 (ref.)	1.08 (0.90–1.30)	1.39 (1.24–1.56)	<0.01
Multivariable-adjusted hazard ratio (95%CI)				
Model 1	1 (ref.)	1.03 (0.86–1.24)	1.34 (1.20–1.50)	<0.01
Model 2	1 (ref.)	1.01 (0.84–1.22)	1.37 (1.22–1.54)	<0.01
Model 3	1 (ref.)	0.98 (0.81–1.18)	1.32 (1.18–1.48)	<0.01

Model 1: adjusted for age, sex, BMI, alcohol intake, regular exercise, diabetes, and dyslipidemia

Model 2: adjusted for variables in model 1 + systolic blood pressure at baseline

Model 3: adjusted for variables in model 1 + amount of weight change from the baseline to the end of study period

We also added an interaction term to the statistical model and conducted a subgroup analysis stratified by potential confounding factors to assess the consistency of the association between smoking status and incident hypertension. After stratification by other risk factors, we estimated the multivariable-adjusted HRs and 95%CIs for the development of hypertension in the comparison of the smoking status to current non-smokers, as shown in Table 3. The factors with/without diabetes and the blood pressure levels were detected as significant interacting factors for the incidence of hypertension. These results suggest that the association between sustained smoking and the incidence of hypertension was stronger in the subjects with lower blood pressure levels (<130/80 mmHg) and in the subjects without diabetes compared to the higher blood pressure levels and the with-diabetes status, respectively.

**Table 3:** Multivariable-adjusted hazard ratios for development of hypertension according to smoking status in subgroups

	Current non-smokers		Quitters		Sustained smokers		P for interaction
	Event/n (%)	HRs	Event/*n* (%)	HRs (95%CI)	Event/*n* (%)	HRs (95%CI)	
Age:							
<40 yrs	231/1905 (12)	ref	58/257 (23)	1.20 (0.90–1.61)	274/1016 (27)	1.45 (1.21–1.74)	0.12
≥40 yrs	364/1128 (32)	ref	85/188 (45)	0.92 (0.73–1.17)	383/945 (41)	1.21 (1.04–1.40)	
Gender							
Male	530/2189 (24)	ref	140/404 (35)	1.04 (0.86–1.25)	639/1852 (35)	1.34 (1.19–1.51)	0.92
Female	65/844 (8)	ref	3/41 (7)	0.83 (0.26–2.67)	18/109 (17)	1.19 (0.68–2.07)	
Overweight/obese							
BMI < 23 kg/m^2^	228/1850 (12)	ref	58/247 (23)	1.09 (0.82–1.47)	209/958 (22)	1.23 (1.01–1.50)	0.69
BMI ≥ 23 kg/m^2^	367/1183 (31)	ref	85/198 (43)	0.98 (0.77–1.25)	448/1003 (45)	1.38 (1.20–1.59)	
Weight gain:							
No	457/2218 (21)	ref	69/238 (29)	0.86 (0.67–1.11)	375/1371 (27)	1.37 (1.20–1.56)	0.15
Yes	138/815 (17)	ref	74/207 (36)	1.28 (0.95–1.72)	282/590 (48)	1.26 (1.00–1.59)	
Blood pressure							
<130/80 mmHg	202/2206 (9)	ref	60/299 (20)	1.34 (0.99–1.80)	267/1244 (21)	1.62 (1.34–1.96)	<0.01
≥130/80 mmHg	393/827 (48)	ref	83/146 (57)	0.86 (0.68–1.09)	390/717 (54)	1.20 (1.04–1.38)	
Regular exercise							
No	400/2016 (20)	ref	102/331 (31)	0.92 (0.74–1.15)	476/1462 (33)	1.25 (1.09–1.43)	0.08
Yes	195/1017 (19)	ref	41/114 (36)	1.31 (0.93–1.85)	181/499 (36)	1.54 (1.26–1.90)	
Alcohol intake							
No	215/1516 (14)	ref	49/157 (31)	1.39 (1.01–1.90)	216/751 (29)	1.40 (1.15–1.71)	0.49
Yes	379/1515 (25)	ref	94/288 (33)	0.90 (0.72–1.13)	439/1207 (36)	1.30 (1.13–1.49)	
Dyslipidemia							
No	269/1992 (13)	ref	47/239 (20)	0.79 (0.57–1.08)	237/954 (25)	1.27 (1.06–1.52)	0.96
Yes	326/1041 (31)	ref	96/206 (47)	1.16 (0.93–1.46)	420/1007 (42)	1.37 (1.18–1.58)	
Diabetes							
No	555/2951 (19)	ref	133/428 (31)	1.05 (0.87–1.27)	627/1881 (33)	1.40 (1.24–1.57)	<0.01
Yes	39/81 (48)	ref	10/17 (59)	0.70 (0.34–1.43)	30/80 (38)	0.60 (0.36–0.98)	

Adjusted for age, sex, body mass index (BMI), alcohol intake, regular exercise, diabetes, and dyslipidemia

In the comparison of the quitters and sustained smokers (Supplementary Table 1), the subjects’ blood pressure levels at baseline and body weight gain during the study period were significant interacting factors. The sensitivity analysis using a logistic regression analysis on participants enrolled from 2007 to 2009 did not make any material difference to the findings (Supplementary Table 2). These results indicate that the risk of new-onset hypertension is reduced in quitters with higher blood pressure (≥130/80 mmHg) and without body weight gain compared to sustained smokers.

In addition, we evaluated the combined effects of weight gain and blood pressure levels on incident hypertension. We compared the hazard ratios for incident hypertension between quitters and sustained smokers, referring to sustained smokers with blood pressure below 130/80 mmHg and without weight gain (Supplementary Table 3). In the groups with weight gain, HRs of quitters were almost same as those of sustained smokers irrespective of blood pressure levels (BP < 130/80 mmHg: sustained smokers 0.91 and quitters 0.95; BP ≥ 130/80 mmHg: sustained smokers 2.35 and quitters 2.46). In contrast, in the groups without weight gain, HRs of quitters were lower compared to those of sustained smokers irrespective of blood pressure levels (BP < 130/80 mmHg: sustained smokers 1.00 and quitters 0.66; BP ≥ 130/80 mmHg: sustained smokers 3.00 and quitters 1.62). However, it was difficult exactly to compare the calculated hazard ratios each other, because the participants in each group were small and it did not reach statistically significant.

## Discussion

The findings revealed by the present analyses of 5439 Japanese workers demonstrated that after multivariable adjustment, the sustained smokers had a significantly higher incidence of hypertension than the non-smokers. The incidence of hypertension in the subjects who quit smoking was as low as that of the non-smokers, especially in the subjects without weight gain during the study period and those with higher blood pressure levels (≥130/80 mmHg) at baseline. These results suggest that smoking cessation could be effective in preventing the development of hypertension, particularly among individuals without weight gain and with higher blood pressure.

The effects of chronic smoking on incident hypertension were divergent in prior investigations. In a > 14.5-year longitudinal study of 13,529 men conducted in the U.S [11]., cigarette smoking was associated with an increase in incident hypertension. This association was also detected in the prospective cohort study of the Women’s Health Study, in which 28,236 women participated and were followed up for 9.8 years [10]. In contrast, in several cohort studies, chronic smoking reduced blood pressure levels and smoking cessation rather increased blood pressure and the incidence of hypertension [18, 28, 29]. The Strong Heart Study reported that 4549 American Indian current smokers did not exhibit an increase in incident hypertension at a 4-year follow-up, but current smoking was inversely related to both SBP and DBP levels [28]. Other cross-sectional studies also showed that current smoking was associated with lower SBP levels [14, 16, 17]. The precise reasons for these divergent results have not been determined, but we speculate that they might be attributable to the study design and heterogeneity of smoking status among the studies. Body weight would be influenced by smoking status (especially after smoking cessation), and the smoking status was not consistent during the study period in some studies [13, 30]. The advantages of the present study are that (i) the subjects’ smoking status information was collected annually over the study period, and (ii) changes in the subjects’ body weight were considered in the evaluation of the development of hypertension.

The strengths of this study were that it included a large number of subjects (>5400) and a long follow-up period (average 6.0 years). The subjects were stratified by potential confounding factors, and then the association between smoking status and the development of hypertension was investigated. In the subgroup analyses, the risk of incident hypertension in the sustained smokers compared to the non-smokers was higher among the subjects without diabetes and those with lower blood pressure levels (<130/80 mmHg) (Table 3), which was consistent with the previous studies [10, 11]. While the exact underlying mechanisms remained unknown in the present study, these results may indicate that the effects of tobacco smoking on incident hypertension is likely to be more prominent among individuals with fewer cardiovascular risk factors. In contrast, the hazard ratio of incident hypertension in the present study’s quitters was lower compared to the sustained smokers with higher blood pressure levels (≥130/80 mmHg). Gaya et al. also showed that blood pressure reduction after smoking cessation was more prominent in the subjects with higher systolic blood pressure (>130 mmHg) at the time of study participation [31]. This finding might indicate that smoking cessation is particularly effective to prevent blood pressure increases in smokers with higher blood pressure levels.

Moreover, in the present study, the quitters without body weight gain showed a lower risk of hypertension development compared to the sustained smokers. We conducted a stratified analysis divided by with/without 3 kg or more weight gain using the weight change from smoking cessation to end of study in quitters, with similar results. Several studies have shown that smoking cessation rather increased the development of hypertension [18, 32]. A recent study conducted in Japan showed that weight gain following smoking cessation led to blood pressure elevation [24], which might support the present findings of maintaining an appropriate body weight after smoking cessation contributes to the prevention of the development of hypertension. In our study, the quitters gained 0.3 kg before and 2.8 kg after smoking cessation, and weight gain of 3 kg or more occurred after smoking cessation in about 70% of them. For those who gained weight after smoking cessation and developed hypertension (55 participants), the average time to weight gain was 0.7 years, and the average time to developing hypertension was 2.8 years from smoking cessation. These results might suggest that weight gain occurred primarily about 1 year after quitting smoking, and that attention should be paid to the development of hypertension for approximately 3 years after smoking cessation.

Considering the combined effects of weight gain and blood pressure levels on the development of hypertension (Supplementary Table 3), smoking cessation reduced the incident hypertension only in the subjects of quitters who did not increase body weight regardless of blood pressure levels. These findings strongly suggest that weight control should be important to prevent the development of hypertension especially after smoking cessation.

We were unable to determine the mechanisms underlying the effect of smoking on the development of hypertension. However, several biological mechanisms have been proposed to explain the relationship between cigarette smoking and hypertension. Acutely, cigarette smoking causes sympathetic activation with an increase in the production of oxidative stress, resulting in an increase in blood pressure [7]. In contrast, chronic smoking may cause endothelial dysfunction [33], vascular injury, plaque progression [34], and increases in insulin resistance [35] and arterial stiffness [36, 37], which could be associated with the development of hypertension [38]. Accordingly, smoking cessation may result in not only a reduction in sympathetic activity but also the recovery of impaired endothelial function and insulin resistance [39–41]. These pathophysiological mechanisms may be involved in the antihypertensive effect of smoking cessation.

Some study limitations should be considered. First, most of the subjects in the study were relatively young and middle-aged Japanese men. It remains unclear whether the present findings can be applied to elderly people, women, or individuals of other ethnicities, although we observed no interaction by gender or age in the stratified analyses. Second, we classified the subjects with a past history of smoking but without smoking at baseline (*n* = 996) as non-smokers, which might have influenced our findings. However, we obtained similar results in a sensitivity analysis excluding these subjects from the non-smoker group (data not shown), which suggests that the influence of these subjects on the results was limited. Third, the smoking status was solely determined by the subjects’ response on a questionnaire, and the smoking status of some of the subjects may thus not have been accurate. Fourth, changes in dietary habits and the precise physical activity of the subjects were not considered. Individuals who tried to quit smoking might be concerned about their health and thus might have reduced their calorie intake and engaged in more physical activity before or after smoking cessation. In fact, the percentage of subjects who improved their exercise or drinking habits was 28% among the current non-smokers, 33% for the quitters, and 20% for the sustained smokers (*p* < 0.05). Lifestyle modifications before or after smoking cessation might therefore contribute to the reduction in the risk of hypertension development in quitters without weight gain. Finally, this study was an observational investigation, and unexpected residual confounding factors might have remained despite our careful covariate adjustment.

### Perspective of Asia

Both smoking and hypertension are independent risk factors for cardiovascular disease [1, 2]. Due to smoking regulations, the global prevalence of smoking tobacco has decreased by 27.5% among men and 37.7% among women over the past 30 years [42]. However, especially among men, smoking prevalence remains still higher in many Asian countries compared to Western countries. While it should be strongly suggested to promote smoking cessation in Asia, maintaining an appropriate body weight after smoking cessation might be important to prevent the development of hypertension.

## Conclusion

Tobacco smoking was associated with an increase in incident hypertension independently of confounding factors. Smoking cessation decreased the incidence of hypertension to the same levels as that of the study’s non-smokers, especially in those with higher levels of blood pressure. Maintaining an appropriate body weight after smoking cessation might also be important to prevent the development of hypertension. Our findings suggest that tobacco smoking is a modest but important risk factor for the development of hypertension, and smoking cessation without weight gain would be effective in the prevention of the development of hypertension.

## Supplementary information

Supplementary Table 1

Supplementary Table 2

Supplementary Table 3
