# Correction: Impact of smoking status on incident hypertension in a Japanese occupational population

**DOI:** 10.1038/s41440-025-02158-3

**Published:** 2025-02-17

**Authors:** Ikumi Yamato, Yasuo Kansui, Kiyoshi Matsumura, Minako Inoue, Ai Ibaraki, Satoko Sakata, Hisatomi Arima, Kenichi Goto, Takanari Kitazono

**Affiliations:** 1 Department of Medicine and Clinical Science, Graduate School of Medical Sciences, Kyushu University, Fukuoka, Japan; 2 Kitakyushu Wakasugi Hospital, Fukuoka, Japan; 3 Center for Cohort Studies, Kyushu University, Fukuoka, Japan; 4 Department of Epidemiology and Public Health, Kyushu University, Fukuoka, Japan; 5 Department of Preventive Medicine and Public Health, Fukuoka University, Fukuoka, Japan; 6 Department of Health Sciences, Kyushu University, Fukuoka, Japan

Correction to: *Hypertension Research*

10.1038/s41440-024-01996-x, published online 08 November 2024

The article “Impact of smoking status on incident hypertension in a Japanese occupational population”, written by Ikumi Yamato, Yasuo Kansui, Kiyoshi Matsumura, Minako Inoue, Ai Ibaraki, Satoko Sakata, Hisatomi Arima, Kenichi Goto, and Takanari Kitazono, was originally published under exclusive license to The Author(s), under exclusive licence to The Japanese Society of Hypertension. As a result of the subsequent decision to publish the article under the open access model, the article’s copyright notice was changed on 5 February 2025 to © The Author(s) 2025 and the article is now distributed under a Creative Commons Attribution 4.0 International License, which permits use, sharing, adaptation, distribution and reproduction in any medium or format, as long as you give appropriate credit to the original author(s) and the source, provide a link to the Creative Commons licence, and indicate if changes were made. The images or other third party material in this article are included in the article’s Creative Commons licence, unless indicated otherwise in a credit line to the material. If material is not included in the article’s Creative Commons licence and your intended use is not permitted by statutory regulation or exceeds the permitted use, you will need to obtain permission directly from the copyright holder. To view a copy of this licence, visit http://creativecommons.org/licenses/by/4.0/.

