# Supplementary material for: Impact of smoking status on incident hypertension in a Japanese occupational population
Source: Hypertens Res. 2024 Nov 8;48(1):180–8. doi: 10.1038/s41440-024-01996-x (PMC11832419; doi:10.1038/s41440-024-01996-x)
Supplement: Supplementary file 1 — Supplementary Table 1 [file 41440_2024_1996_MOESM1_ESM.docx]

**Supplementary Table 1.** Multivariable-adjusted hazard ratios for development of hypertension in quitters (vs sustained smokers)

|  |  | **Quitters (vs. Sustained smokers)** | |
| --- | --- | --- | --- |
|  |  | **HR (95%CI)** | **P for interaction** |
| Age: | <40 yrs | 0.81 (0.61–1.08) | 0.73 |
|  | ≥40 yrs | 0.76 (0.60–0.97) |  |
| Gender: | Male | 0.78 (0.65–0.93) | 0.73 |
|  | Female | 0.56 (0.16–2.01) |  |
| Overweight/obese: | BMI <23 kg/m^2^ | 0.88 (0.66–1.18) | 0.43 |
|  | BMI ≥23 kg/m^2^ | 0.71 (0.56–0.90) |  |
| Weight gain: | No | 0.63 (0.49–0.81) | <0.01 |
|  | Yes | 1.05 (0.79–1.38) |  |
| Blood pressure: | <130/80 mmHg | 0.83 (0.63–1.10) | <0.01 |
|  | ≥130/80 mmHg | 0.70 (0.55–0.90) |  |
| Regular exercise: | No | 0.73 (0.59–0.91) | 0.40 |
|  | Yes | 0.86 (0.61–1.21) |  |
| Alcohol intake: | No | 0.97 (0.71–1.32) | 0.10 |
|  | Yes | 0.70 (0.56–0.87) |  |
| Dyslipidemia: | No | 0.63 (0.46–0.87) | 0.21 |
|  | Yes | 0.84 (0.67–1.05) |  |
| Diabetes: | No | 0.75 (0.62–0.91) | 0.19 |
|  | Yes | 1.36 (0.64–2.89) |  |

Adjusted for age, sex, body mass index (BMI), alcohol intake, regular exercise, diabetes, and dyslipidemia.
