# Supplementary material for: Impact of smoking status on incident hypertension in a Japanese occupational population
Source: Hypertens Res. 2024 Nov 8;48(1):180–8. doi: 10.1038/s41440-024-01996-x (PMC11832419; doi:10.1038/s41440-024-01996-x)
Supplement: Supplementary file 2 — Supplementary Table 2 [file 41440_2024_1996_MOESM2_ESM.docx]

**Supplementary Table 2.** Multivariable-adjusted odds ratios for development of hypertension in quitters (n=387) (vs sustained smokers n=1,521), who enrolled in the study from 2007 to 2009

|  |  | **Quitters (vs. Sustained smokers)** | |
| --- | --- | --- | --- |
|  |  | **Odds ratio (95%CI)** | **P for interaction** |
| Age: | <40 yrs | 0.89 (0.61–1.30) | 0.39 |
|  | ≥40 yrs | 1.03 (0.73–1.44) |  |
| Gender: | Male | 0.98 (0.76–1.26) | 0.44 |
|  | Female | 0.56 (0.11–2.89) |  |
| Overweight/obese: | BMI <23 kg/m^2^ | 1.12 (0.78–1.62) | 0.53 |
|  | BMI ≥23 kg/m^2^ | 0.83 (0.60–1.16) |  |
| Weight gain: | No | 0.68 (0.49–0.95) | <0.01 |
|  | Yes | 1.35 (0.90–2.02) |  |
| Blood pressure: | <130/80 mmHg | 1.00 (0.71–1.41) | <0.01 |
|  | ≥130/80 mmHg | 0.96 (0.65–1.42) |  |
| Regular exercise: | No | 0.93 (0.70–1.24) | 0.58 |
|  | Yes | 1.08 (0.66–1.76) |  |
| Alcohol intake: | No | 1.31 (0.85–2.02) | 0.14 |
|  | Yes | 0.84 (0.62–1.14) |  |
| Dyslipidemia: | No | 0.72 (0.48–1.06) | 0.06 |
|  | Yes | 1.18 (0.85–1.65) |  |
| Diabetes: | No | 0.91 (0.71–1.18) | 0.04 |
|  | Yes | 4.52 (1.22–16.8) |  |

Adjusted for age, sex, body mass index (BMI), alcohol intake, regular exercise, diabetes, and dyslipidemia.
