# Supplementary material for: Impact of smoking status on incident hypertension in a Japanese occupational population
Source: Hypertens Res. 2024 Nov 8;48(1):180–8. doi: 10.1038/s41440-024-01996-x (PMC11832419; doi:10.1038/s41440-024-01996-x)
Supplement: Supplementary file 3 — Supplementary Table 3 [file 41440_2024_1996_MOESM3_ESM.docx]

**Supplementary Table 3.** Multivariable-adjusted hazard ratios for development hypertension between quitters and sustained smokers according to blood pressure level and with/without weight gain.

|  |  | Weight gain (-) | |  | Weight gain (+) | |
| --- | --- | --- | --- | --- | --- | --- |
|  |  | Sustained smokers | Quitters |  | Sustained smokers | Quitters |
| BP<130/80　mmHg |  | ref | 0.66  (0.44-1.01) |  | 0.91  (0.70-1.17) | 0.95  (0.66-1.37) |
| BP≥130/80　mmHg |  | 3.00  (2.48-3.48) | 1.62  (1.16-2.26) |  | 2.35  (1.81-3.07) | 2.46  (1.74-3.48) |

Adjusted for age, sex, body mass index (BMI), alcohol intake, regular exercise, diabetes, and dyslipidemia.
